# Characteristics of the Exradin W1 scintillator in the magnetic field

**DOI:** 10.1002/acm2.12707

**Published:** 2019-08-28

**Authors:** Jeongmin Yoon, Jung‐In Kim, Chang Heon Choi, Jong Min Park

**Affiliations:** ^1^ Department of Radiation Oncology Seoul National University Hospital Seoul Korea; ^2^ Institute of Radiation Medicine Seoul National University Medical Research Center Seoul Korea; ^3^ Biomedical Research Institute Seoul National University College of Medicine Seoul Korea; ^4^ Robotics Research Laboratory for Extreme Environments Advanced Institutes of Convergence Technology Suwon Korea

**Keywords:** angular dependency, magnetic resonance image‐guided radiation therapy, output factor, plastic scintillation detector

## Abstract

To investigate the angular dependency of the W1 scintillator with and without a magnetic field, the beam incidence angles to the detector varied from 0° to 360° at intervals of 30° when the detector was pointed in both the craniocaudal and right‐to‐left directions. The beam incidence angles also varied from 0° to 360° at intervals of 45° when the W1 scintillator was in the anterior‐to‐posterior direction. To investigate the field size dependency of the W1 scintillator with and without a magnetic field, the doses by an identical beam‐on time were measured at various square field sizes and the measured doses were normalized to the dose at the field of 10.5 cm × 10.5 cm (FS10.5). With and without a magnetic field, the deviations of the doses to the dose at the beam incident angle of 0° were always less than 1% regardless of the dosimeter positioning relative to the magnetic field direction. When the field sizes were equal to or less than FS10.5, the differences in the output factors with and without a magnetic field were less than 0.7%. However, those were larger than 1% at fields larger than FS10.5, and up to 3.1%. The W1 scintillator showed no angular dependency to the magnetic field. Differences larger than 1% in the output factors with and without a magnetic field were observed at field sizes larger than 10.5 cm × 10.5 cm.

## 1 INTRODUCTION

Magnetic resonance image‐guided radiation therapy (MR‐IGRT) became available in the field of radiation therapy with the release of the ViewRay™ system (ViewRay Inc.).^1^ Since magnetic resonance imaging (MRI) provides superior soft tissue contrast compared to that of CT imaging, more accurate delineations of the target volumes for liver cancer, prostate cancer, and brain tumors are possible.^2^, ^3^, ^4^ Moreover, MRI does not deliver an imaging dose to the patient, therefore, daily imaging for verification of patient setup, or imaging during treatment for the monitoring of patient respiratory motion, are not limited.^5^ This facilitates adaptive radiation therapy (ART) based on daily 3‐D image sets, as well as respiratory‐gated radiation therapy based on real‐time patient's internal anatomy motion.^5^ Therefore, accurate and precise treatment reflecting actual tumor shape, location, and motion is feasible with MR‐IGRT on a daily basis. Moreover, the ART and respiratory‐gated radiation therapy based on a patient's internal anatomy enable the reduction of the target margin which compensates for both the inter‐ and intra‐fractional errors.^3^, ^5^ Reduction of the target margins results in the sparing of doses near the target volume, which is highly desirable in order to minimize radiotherapy‐induced complications.^6^, ^7^, ^8^, ^9^ Furthermore, dose escalation can be achieved by the reduction of the target margins with MR‐IGRT since a limit of dose escalation is to maintain the delivered doses to organs at risk near the target volume to be lower than the normal tissue tolerance level.^8^, ^10^ Therefore, MR‐IGRT is promising to improve the accuracy of radiation therapy and to enhance treatment efficacy while reducing treatment‐related complications.

Although MR‐IGRT is a promising treatment technique, the presence of the magnetic field may lower the accuracy of the dosimeters utilized in the field of radiation therapy.^11^, ^12^ Various studies have investigated the behaviors of existing dosimeters for radiation therapy in the presence of a magnetic field and have performed the development of new dosimeters compatible with a magnetic field.^13^, ^14^, ^15^, ^16^ As an existing dosimeter for radiation therapy, the organic plastic scintillation detector (PSD) showed an outstanding performance for small‐field dosimetry and in vivo dosimetry owing to its small dimensions and near‐water equivalence of composing materials.^17^, ^18^ However, Strefanowicz et al. reported an increase of 7% in the light intensity of PSDs by a magnetic field of 1 T.^19^ Therriault‐Proulx et al. also investigated the response of PSDs by a magnetic field by varying the magnetic field strength from 0 T to 1.5 T.^20^ They found that the increase in the light intensity of the PSDs by a magnetic field is mainly due to the Cerenkov effects. Simiele et al. also reported that the changes in the light intensities of PSDs by a magnetic field were attributed to the directional nature of Cerenkov light emission.^21^ Several studies have investigated the behavior of PSDs in a magnetic field, however, these focused on the light intensity variations according to the strength of the magnetic field.^20^, ^21^ As yet, no studies have been performed to investigate the angular dependency or field size dependency of PSDs in a magnetic field. To utilize PSDs for dosimetry in a magnetic field, these characteristics should be investigated.

In this study, we investigate the changes in the angular dependency as well as field size dependency of PSDs in a magnetic field. In our institution, we had a rare opportunity to shut down the magnetic field of the ViewRay system for maintenance purposes. Therefore, we were able to investigate the responses of the PSDs using identical beams with and without a magnetic field. We investigated the angular dependency and field size dependency of the PSDs in the magnetic field at various orientations relative to the magnetic field direction compared to those without the magnetic field.

## 2 MATERIALS AND METHODS

### 2.1 Irradiation condition of plastic scintillation detector

To investigate the characteristics of PSDs with and without a magnetic field, we irradiated the PSDs with the ViewRay system, which utilizes a total of 3 Co‐60 radiation sources (head 1, 2, and 3).^1^ Since the magnetic field strength of the ViewRay system is 0.35 T, the PSDs were tested in this study with and without the 0.35‐T magnetic field of the ViewRay system.^22^ For both situations with and without the magnetic field, we performed reference dosimetry following the American Association of Physicists in Medicine (AAPM) Task Group 51 (TG‐51) protocol to verify the delivery time for a 1 Gy delivery, calculated by the treatment planning system (TPS), i.e., the MRIdian™ system (ViewRay Inc.).^23^ The reference doses with and without the magnetic field were measured with a MR‐compatible Exradin A12 ionization chamber (Standard Imaging), connected to a UNIDOS^®^ E electrometer (PTW) in a water phantom.

### 2.2 Calibration of the PSD

**Figure 1:**
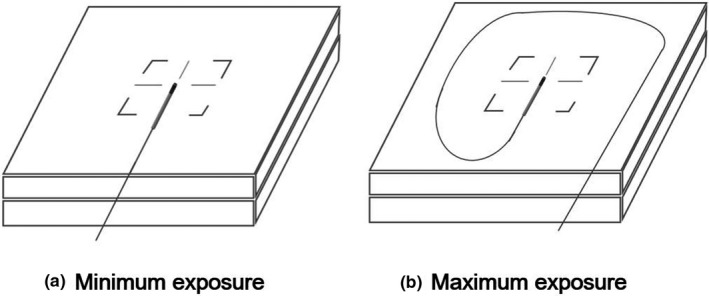
Two configurations of the (a) minimum and (b) maximum exposures of the optic fibers for the Cerenkov effect correction. For the Cerenkov effect correction, calibration slabs provided by the manufacturer were used.

The Exradin W1 scintillator (Standard Imaging), an organic PSD, was tested in this study. The scintillating fiber of the W1 scintillator was polystyrene with an acrylonitrile butadiene styrene (ABS) plastic enclosure and polymide stem.^18^ The dimensions of the W1 scintillator were 1 mm in diameter and 3 mm in length. The W1 scintillator was connected to a 2‐channel SuperMAX electrometer with a 3 m‐long optic fiber (Standard Imaging). The correction of the Cerenkov light to the signal of the W1 scintillator was performed as recommended by the manufacturer, which was the spectral method originally proposed by Guillot et al.^24^ For the Cerenkov effect correction, we used the Exradin scintillator calibration slabs (Standard Imaging) at the two configurations of the minimum and maximum exposures of the optic fibers, as shown in Fig. 1. For the maximum exposure of the optic fiber, the field size recommended by the manufacturer was 40 cm × 40 cm.^1^ However, since the maximum field size of the ViewRay system was 27.3 cm × 27.3 cm at the isoplane, we measured the signals of the W1 scintillator for the maximum exposure at the field size of 27.3 cm × 27.3 cm instead of 40 cm × 40 cm. According to a previous study by Carrasco et al. on the characteristics of the W1 scintillator, the effect of changing the length of the optic fiber in the field (with a field size of 25 cm × 25 cm) on the values of the Cerenkov light ratio was minimal.^18^ Therefore, we used the field size of 27.3 cm × 27.3 cm for the maximum exposure of the optic fiber in this study. The calibrations of the W1 scintillator were performed for both situations, with and without the magnetic field, respectively.

### 2.3 Angular dependency of the PSD

**Figure 2:**
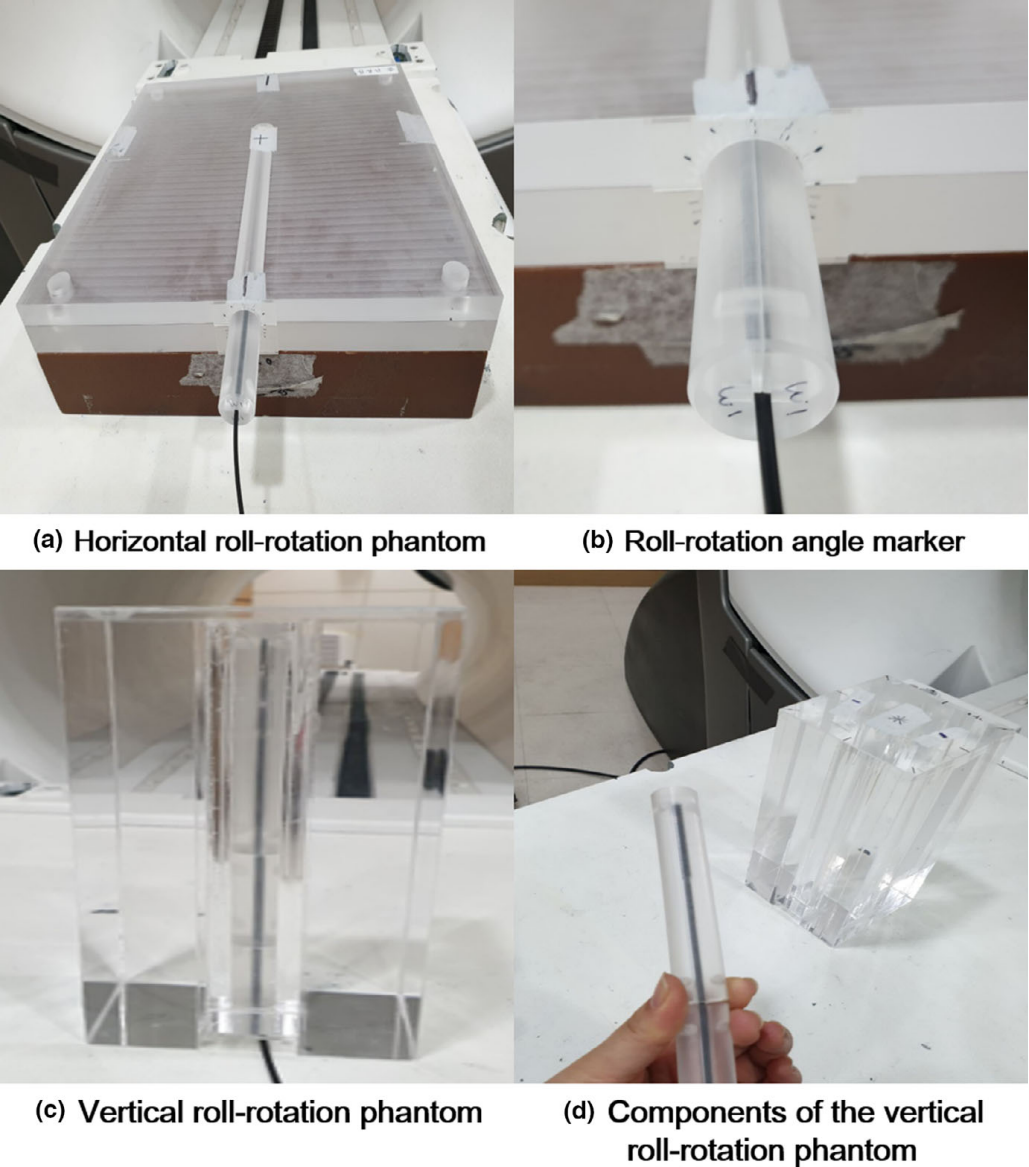
(a) A custom‐made acrylic phantom that can locate the W1 scintillator parallel to the couch surface (horizontal roll‐rotation phantom). (b) Incidence angle of the beam to the W1 scintillator can be manually adjusted by rotating an acrylic cylinder inside the horizontal roll‐rotation phantom. (c) A custom‐made acrylic phantom which can locate the W1 scintillator vertical to the couch surface (vertical roll‐rotation phantom). (d) Like the acrylic cylinder of the horizontal roll‐rotation phantom, an acrylic cylinder can be inserted in the vertical roll‐rotation phantom to adjust angles between the W1 scintillator and the magnetic field direction.

For irradiation of the W1 scintillator in this study, we used only head 3 of the ViewRay system, which covers gantry angles from 270° to 30° in the treatment mode.^1^ The W1 scintillators were always irradiated by 1 Gy at a gantry angle of 0°. Every measurement in this study was repeated three times and the average values of the three measurements were evaluated. With and without the magnetic field, we measured the Cerenkov‐light‐corrected readings of the W1 scintillators at various angles between the beam direction and the W1 scintillator by utilizing custom‐made acrylic phantoms, as shown in Fig. 2.^24^ The W1 scintillator was located parallel to the couch surface in the custom‐made acrylic phantom (termed horizontal roll‐rotation phantom) [Figs. 2(a) and 2(b)]. With the horizontal roll‐rotation phantom, the incidence angle of the beam to the W1 scintillator could be manually adjusted by roll‐rotating an acrylic cylinder inside the horizontal roll‐rotation phantom. In addition, the W1 scintillator was located vertical to the couch surface, i.e., parallel to the beam direction, in the other custom‐made acrylic phantom (termed vertical roll‐rotation phantom) [Figs. 2(c) and 2(d)]. With the vertical roll‐rotation phantom, the angles between the W1 scintillator and the magnetic field direction could be manually adjusted by roll‐rotating an acrylic cylinder inside the vertical roll‐rotation phantom.

**Figure 3:**
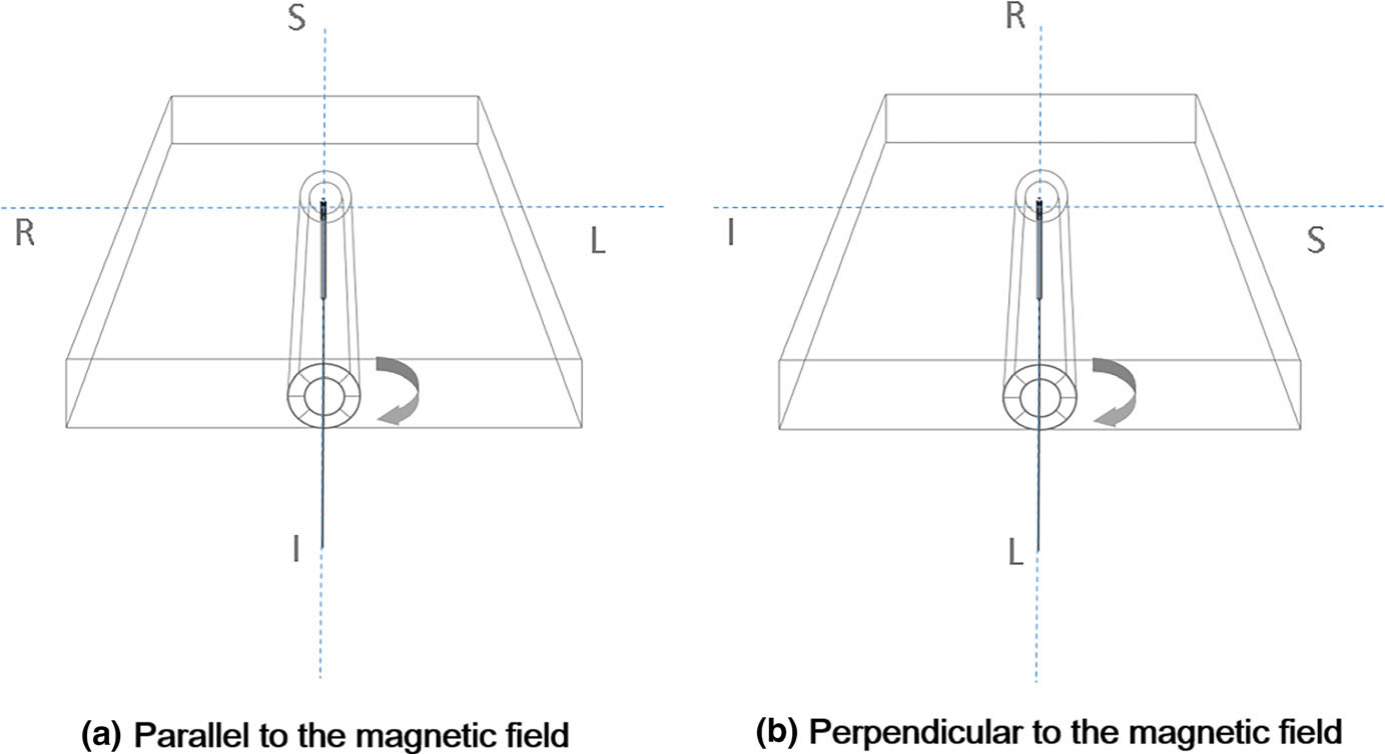
With the horizon roll‐rotation phantom, doses of 1 Gy were measured with the W1 scintillator while rotating it from 0° to 360° at intervals of 30°. (a) When measuring doses, the W1 scintillator was located parallel to the magnetic field direction as well as orthogonal to the beam direction. (b) We repeated the measurements positioning the W1 scintillators orthogonal to both the magnetic field direction and the beam direction.

With the horizontal roll‐rotation phantom and solid water phantoms, we measured doses of 1 Gy at both 1.5 cm and 5 cm depths while rotating the W1 scintillator from 0° to 360°, using the international electrotechnical commission (IEC) 1217 coordinate system, at intervals of 30°, positioning the W1 scintillator parallel to the magnetic field direction, i.e., craniocaudal (CC) direction, when the patient position was head first supine (HFS) [Fig. 3(a)]. Since the shape of the W1 scintillator was symmetric, an arbitrary position was designated as a reference position and we roll‐rotated the W1 scintillator by rotating the cylinder with marking in the horizontal roll‐rotation phantom. The W1 scintillator was always located at the isoplane (105 cm from the source), therefore, the source‐to‐surface distances (SSDs) at 1.5 and 5 cm depths were 103.5 and 100 cm, respectively. In addition, we also measured doses of 1 Gy at both 1.5 and 5 cm depths while roll‐rotating the W1 scintillators from 0° to 360° at intervals of 30°, positioning the W1 scintillator orthogonal to both the magnetic field direction and the beam direction, i.e., right‐to‐left (RL) direction at the HFS position [Fig. 3(b)]. Similar to the CC direction setup, the W1 scintillator was always located at the isoplane in the case of RL direction setup. The measurements without the magnetic field were performed only in the CC direction when using the horizontal roll‐rotation phantom, i.e., no measurements in the RL direction were performed without the magnetic field since there is no difference between the measurements in the CC and RL direction when there is no magnetic field.

**Figure 4:**
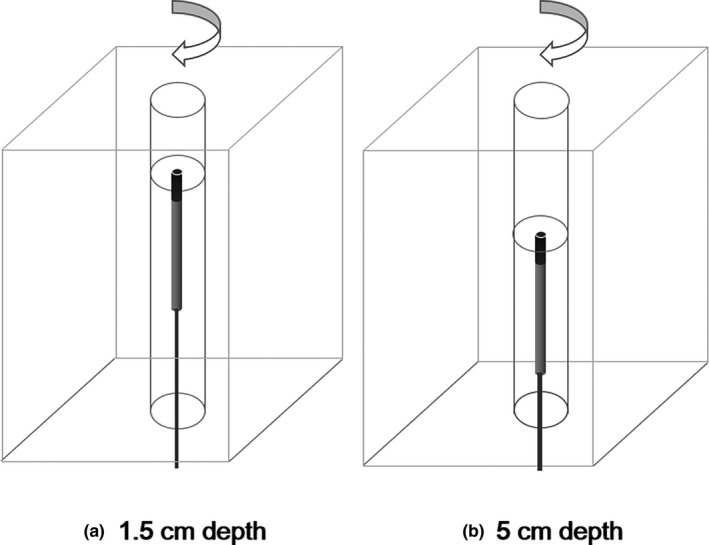
With the vertical roll‐rotation phantom, doses of 1 Gy were measured with the W1 scintillator while rotating it from 0° to 360° at intervals of 45°, positioning the W1 scintillator parallel to the beam direction. The measurements were performed at depths of (a) 1.5 cm and (b) 5 cm.

With the vertical roll‐rotation phantom, we measured doses of 1 Gy at both 1.5 and 5 cm depths while rotating the W1 scintillator from 0° to 360° at intervals of 45°, positioning the W1 scintillator parallel to the beam direction, i.e., anterior‐to‐posterior (AP) direction at the HFS position (Fig. 4). The center of the W1 scintillator was always located at the isoplane.

For each measurement at the setup of the W1 scintillators, along the CC, RL, and AP directions, measured doses were normalized to the values taken at 0°, i.e., the value at the reference position. Every measurement was performed with and without the magnetic field and compared to each other.

### 2.4 Field size dependency

With the Exradin scintillator calibration slabs (minimum exposure) and solid water phantoms, we measured doses of an identical beam‐on time (beam‐on time delivering 1 Gy when the field size was 10.5 cm × 10.5 cm) using the W1 scintillator at depths of 1.5 and 5 cm with various square field sizes. The field size dimensions were 4.2, 6.3, 8.4, 10.5, 12.6, 14.7, 16.8, 21.0, and 27.3 cm (a total of nine field sizes) at the isoplane. The W1 scintillator was always located at the isoplane (105 cm from the source), therefore, the SSDs at 1.5 and 5 cm depths were 103.5 and 100 cm, respectively. The measured doses were normalized to the dose with the field size of 10.5 cm × 10.5 cm. Every measurement was performed with and without the magnetic field and compared to each other.

## 3 RESULTS

The doses measured with the W1 scintillator showed high reproducibility, always showing standard deviations of less than 0.3% of the three repeated measurements.

### 3.1 Angular dependency of the PSDs with and without the magnetic field

**Figure 5:**
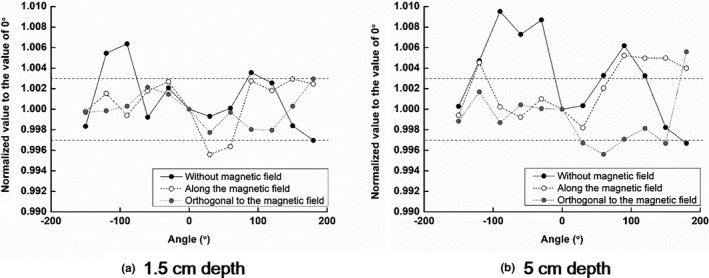
With the horizontal roll‐rotation phantom, the doses were measured with the W1 scintillator normalized to the measured dose at 0° (reference position) at various beam incident angles. The measured values are given at depths of (a) 1.5 cm and (b) 5 cm, with and without the magnetic field.

With the horizontal roll‐rotation phantom, the measured values at the various angles between the W1 scintillator and the beam direction, normalized to the measured dose at 0° (reference position), are plotted in Fig. 5.

At the depth of 1.5 cm, without the magnetic field, the maximum percent deviation from the dose at the reference position was 0.6% at the angle of 270°. The average normalized value was 1.001 ± 0.003, showing no angular dependency. When the W1 scintillators were located at the CC and RL directions with the magnetic field present, the maximum percent deviations were − 0.4% (at 60°) and 0.3% (at 180°), respectively. The average normalized values at the CC and RL directions were 1.001 ± 0.002 and 1.000 ± 0.002, respectively, showing no angular dependencies.

At the depth of 5 cm, without the magnetic field, the maximum percent deviation from the dose at the reference position was 1.0% at the angle of 270°. The average normalized value was 1.003 ± 0.004 showing no angular dependency. When the W1 scintillators were located at the CC and RL directions with the magnetic field, the maximum percent deviations were 0.5% (at 120°) and 0.6% (at 180°), respectively. The average normalized values at the CC and RL directions were 1.002 ± 0.003 and 0.999 ± 0.003, respectively, showing no angular dependencies.

**Figure 6:**
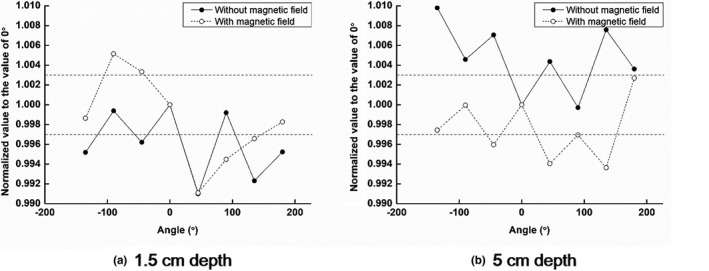
With the vertical roll‐rotation phantom, the measured doses at various angles between the W1 scintillator and the magnetic field direction, normalized to the measured dose at 0° (reference position) are plotted at depths of (a) 1.5 cm and (b) 5 cm, with and without the magnetic field.

With the vertical roll‐rotation phantom, the measured values at the various angles between the W1 scintillator and the magnetic field direction, normalized to the measured dose at 0° (reference position), are plotted in Fig. 6.

At the depth of 1.5 cm, without the magnetic field, the maximum percent deviation from the dose at the reference position was − 0.9% at the angle of 45°. The average normalized value was 0.996 ± 0.003, showing no angular dependency. With the magnetic field, the maximum percent deviation was − 0.9% (at 45°). The average normalized value was 0.998 ± 0.004, showing no angular dependency.

At the depth of 5 cm, without the magnetic field, the maximum percent deviation from the dose at the reference position was 1.0% at the angle of 315°. The average normalized value was 1.005 ± 0.003, showing no angular dependency. With the magnetic field, the maximum percent deviation was − 0.6% (at 315°). The average normalized value was 0.998 ± 0.003, showing no angular dependency.

### 3.2 Field size dependency of the PSDs with and without the magnetic field

**Figure 7:**
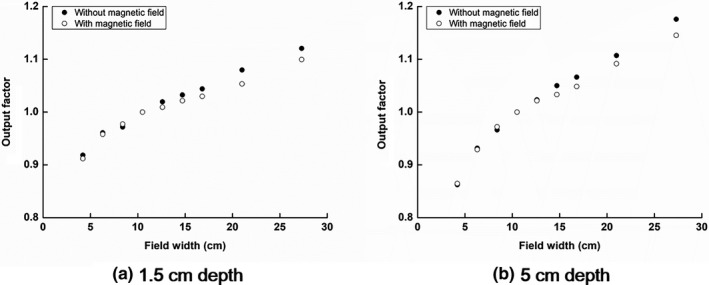
Output factors at various field sizes at depths of (a) 1.5 cm and (b) 5 cm, with and without the magnetic field.

The output factors with and without the magnetic field are shown in Fig. 7. At both the depths of 1.5 and 5 cm, the differences between the output factors with and without the magnetic field were less than 0.7% at field sizes equal to or less than 10.5 cm × 10.5 cm. However, the output factors with field sizes equal to or larger than 12.6 cm × 12.6 cm, at the depth of 1.5 cm with the magnetic field, showed differences larger than 1% compared to those without the magnetic field. The maximum difference between the output factors with and without the magnetic field was 2.6% when the field size was 21.0 cm × 21.0 cm. At the 5 cm depth, the output factors with field sizes equal to or larger than 14.7 cm × 14.7 cm showed differences larger than 1% compared to those without the magnetic field. The maximum difference between the output factors with and without the magnetic field was 3.1% at the field size of 27.3 cm × 27.3 cm.

## 4 DISCUSSION

The organic PSD has advantages in dosimetry for radiation therapy including real‐time measurement, water equivalence of composing materials, energy‐independency for clinical megavoltage radiations, and high resolution measurement owing to its small dimensions.^17^ In order to utilize organic PSDs for the emerging radiation therapy technique of MR‐IGRT, the dosimetric characteristics of organic PSDs in a magnetic field should be thoroughly investigated. Therefore, in the present study, we investigated the effects of a magnetic field on the angular dependency as well as the field size dependency of a W1 scintillator, an organic PSD, by measuring doses using the W1 scintillator with and without a magnetic field (0.35 T magnetic field strength of the ViewRay system used in this study). The doses were measured using the W1 scintillator under identical conditions, except for the presence of the magnetic field, to control other factors that may cause differences in the measured doses. Since the performance of the W1 scintillator as a dosimeter without a magnetic field has already been validated in previous studies,^17^, ^18^ we regarded the measured doses by the W1 scintillator without the magnetic field as the reference values. Without the magnetic field, the W1 scintillator did not show angular dependency, with deviations of less than 1% of the measured values at various beam incident angles; the same as the results of a previous study.^18^ With a magnetic field of 0.35 T in strength, no angular dependencies were observed in the CC (parallel to the magnetic field), RL (perpendicular to the magnetic field and parallel to the beam), and AP directions (perpendicular to both the magnetic field and beams), showing deviations of less than 1% in the measured values at various beam incident angles. On the contrary, the output factors measured with the W1 scintillator in the magnetic field, which represent field size dependency, were different from those without the magnetic field, showing differences of more than 1% and up to 3.1% when the field sizes were larger than 10.5 cm × 10.5 cm. There was a tendency in which the measured doses at the larger field sizes of the magnetic field were smaller than those without the magnetic field, i.e., an underestimation of doses with the W1 scintillator in the magnetic field was observed. Therefore, dosimetry with the W1 scintillator for MR‐IGRT at large field sizes (>10.5 cm × 10.5 cm) does not seem appropriate and some correction factors should be applied.

In the present study, we performed reference dosimetry following the AAPM TG‐51 protocol with and without the magnetic field. Although the AAPM TG‐51 protocol is not designed to be used in the magnetic field, we applied it in the magnetic field since several studies demonstrated successful applications of the AAPM TG‐51 protocol for the reference dosimetry in the 0.35‐T magnetic field.^25^, ^26^ In addition, we used MR‐compatible ionization chamber to perform reference dosimetry in the magnetic field. Similar to the results of the previous studies, we found that the difference in the doses obtained following the AAPM TG‐51 protocol with and without magnetic field was negligible, which was only 0.5% (data are not shown).^25^, ^26^

The standard deviations of the repeated W1 scintillator measurements with identical setup, with and without the magnetic field, were always less than 0.3%, showing high reproducibility. Therefore, the presence of a magnetic field does not affect the measurement reproducibility of the W1 scintillator.

Although we observed a phenomenon of dose underestimation measured with the W1 scintillator in the magnetic field at large field sizes in this study, we did not determine its reason. Since portions of optic fiber inclusions in the large fields were larger than those at the small fields, we speculate that the Cerenkov light correction in the magnetic field might be different from that without the magnetic field. Further investigation of the reason for the deviations observed at the large field sizes of the W1 scintillator readings will be conducted in the future. In addition, in this study, we did not investigate the dose linearity, dose rate dependency, nor changes in the reading according to the accumulated dose of the W1 scintillator in the magnetic field. This will also be completed in the future.

The results in this study are only valid for the 0.35‐T magnetic field of the ViewRay system or the MRIdian^®^ Linac (ViewRay Inc.). The characteristics of the W1 scintillator with the Elekta Unity (Elekta AB) would not be the same as shown in this study since the magnetic field strength of the Elekta Unity is higher than that of the ViewRay system or the MRIdian Linac, which is 1.5 T. This is a limitation of the present study.

## 5 CONCLUSIONS

The W1 scintillator showed no angular dependency regardless of the dosimeter allocation relative to the magnetic field direction, therefore, no consideration of the dosimeter direction is necessary when performing dosimetry with a W1 scintillator for MR‐IGRT. At the field sizes equal to or less than 10.5 cm × 10.5 cm, no effect of the magnetic field on the readings of the W1 scintillator was observed. However, at the large field sizes, larger than 10.5 cm × 10.5 cm, the measured doses in the magnetic field were smaller than those without the magnetic field, being up to 3.1%. Hence, we do not recommended the use of a W1 scintillator at field sizes larger than 10.5 cm × 10.5 cm with the magnetic field.

## CONFLICT OF INTEREST

No conflict of interest.
